# Refractive Lens Exchange Combined with Primary Posterior Vitrectorhexis in Highly Myopic Patients

**DOI:** 10.1155/2017/7826735

**Published:** 2017-04-20

**Authors:** Tarek A. Mohamed, Wael Soliman, Dalia M. EL Sebaity, Ahmed M. Fathalla

**Affiliations:** Ophthalmology Department, Assiut University Hospital, Assiut, Egypt

## Abstract

*Purpose*. To evaluate efficacy and safety of primary vitrectorhexis for posterior capsulotomy in highly myopic patients undergoing refractive lens exchange. *Methods*. The study is a prospective nonrandomized interventional study. The study comprised 60 eyes of 60 myopic patients. All patients underwent refractive lens exchange (RLE) and foldable IOL implantation combined with primary posterior capsulotomy. We used a 23-gauge vitrectomy probe for the creation of the posterior capsule opening. We followed the patients for one year. *Results*. During surgery, the IOLs remained well centered in the capsular bag after creation of the capsulotomy. Postoperatively, we did not report any complications related to lens centration or changes in the posterior capsulotomy size. No eye required YAG laser posterior capsulotomy and no cases of retinal detachment (RD) occurred during the follow-up period. *Conclusion*. Primary posterior vitrectorhexis during RLE is an efficient method in preventing the occurrence of posterior capsular opacification (PCO) and the need for YAG laser posterior capsulotomy with its possible complications.

## 1. Introduction

Myopic patients are often willing to have refractive surgery to be independent from contact lenses or spectacles [1]. The concept of RLE dates back to the eighteenth century. However, this procedure was gradually abandoned due to high rates of postoperative retinal detachment [2, 3]. The development of new concepts and techniques in lens surgery in the 20th century led to a renaissance of RLE [2].

The presence of a high refractive error, which may be associated with an abnormal ocular anatomy, high patient expectations, and the clarity of crystalline lens make RLE different from standard cataract surgery [4]. Posterior capsular opacification (PCO) is a one of the most common complications of the RLE procedure especially in those relatively young age patients. PCO can develop months to years after surgery and can be managed by YAG capsulotomy to reverse its vision-disabling effect [5]. YAG capsulotomy is associated with significant anterior and posterior segment complications such as IOP elevation, IOL pitting, uveitis, cystoid macular edema (CME), and retinal detachment (RD) [6, 7]. The term vitrectorhexis was first used by Wilson et al. in 1999 [8] who described a technique that used a vitrector hand piece to perform capsulotomy in pediatric cataract surgery [8, 9]. To the best of our knowledge, there is no reported series investigating the technique of vitrectorhexis for posterior capsulotomy in adults. The aim of this study was to evaluate efficacy and safety of vitrectorhexis for posterior capsulotomy in myopic adult patients undergoing RLE.

## 2. Patients and Methods

This prospective interventional study comprised 60 eyes of 60 myopic patients (22 males and 38 females). Surgeries were done during the period between May 2014 and March 2015. The study was conducted after getting the agreement of the Ethics Committee at the Faculty of Medicine, Assiut University. The study followed the Declaration of Helsinki. Patients were seeking refractive surgery due to either refusal of glasses or intolerance to contact lenses. Those patients were not LASIK candidates due to high refractive error or thin cornea. Informed consents were obtained from all patients. Preoperative evaluation included UCVA, BCVA (decimal notation), applanation tonometry, B-scan ultrasonography, and detailed fundus examination with particular attention to the retinal periphery. Prophylactic retinal argon laser photocoagulation was performed for any suspicious areas at least 2 weeks prior to surgery using 532 nm green laser (Integre Pro, Ellex). Biometry was done using the optical biometry device (AL-Scan, NIDEK).

Operations were carried out under local anesthesia by the same surgeon. Two side-port incisions (1.0 mm) were created in the clear cornea. The anterior chamber was filled with sodium hyaluronate. We performed anterior capsulorhexis through one of the paracenteses. A 2.2 mm main clear corneal incision was done. After evacuation of some viscoelasticity, we performed hydrodissection through the main incision. Subsequently, either phacoaspiration or bimanual irrigation aspiration was performed. Then, any residual cortical material was removed using bimanual irrigation aspiration. We used sodium hyaluronate (Healon) to reform the anterior chamber and inflate the capsular bag. Then, we implanted a single-piece hydrophilic acrylic intraocular lens (Freedom Fold HFC-603) in the capsular bag. After IOL implantation, posterior capsulotomy using the vitrectomy probe (23 gauge) was done through the main incision after inflation of the capsular bag and anterior chamber with sodium hyalauronate 1% (Healon). With the irrigation cannula in the anterior chamber, the vitrectomy probe was introduced behind the IOL with port directed posteriorly (port down). Then, we removed the irrigation cannula to avoid vitreous hydration and subsequent increase in IOP. With a cutting rate of 500–600 cuts per minute and vacuum of 100–150 mmHg, a small opening was created in the posterior capsule. This was followed by turning the port of the cutter anteriorly (port up) to enlarge the opening to a size of about 4.00 mm. With the cutter off, we brought the instrument in the anterior chamber and IOL optic manipulated back in the bag. Then, the instruments were withdrawn from the eye. Stromal hydration of the side and main ports were done. Any residual sodium hyaluronate was removed from the eye (see Video 1 available online at https://doi.org/10.1155/2017/7826735).

Postoperatively, all patients were treated with 1% topical prednisolone acetate eye drops 5 times daily and tapered over 4 weeks and 0.5% moxifloxacin four times daily for 4 weeks.

Patients were followed up at day one and after one week, one month, 6 months, and one year. Examination was done to assess any intraocular inflammation, IOL centration, IOP, and patency of the posterior capsule opening. Detailed fundus examination was done using indirect ophthalmoscopy. Most of our patients became spectacle-independent and they depend on their corrected eye. However, contact lens was prescribed to some of them. This was until the other eye was managed by RLE at our center or elsewhere.

We analyzed the results using the SPSS computer software package, version 10.0 (Chicago, IL, USA). Continuous data were shown as means with standard deviation. For comparison, we used the paired *t*-test. All tests were considered statistically significant at *P* > 0.05. Normality of the data was checked using Kolmogorov-Smirnov test.

### 2.1. Results

**Figure 1:**
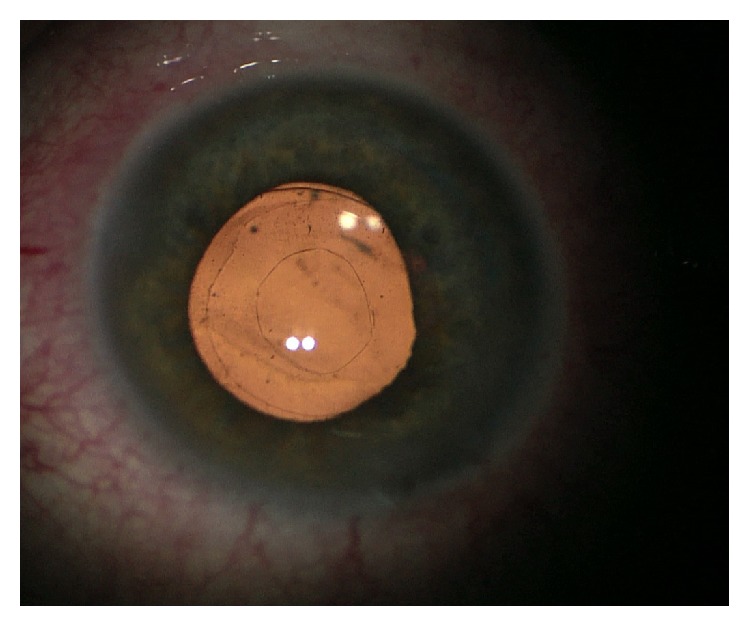
Well-defined patent posterior capsulotomy (vitrectorhexis) with a well-centralized IOL.

Mean age was 36.46 ± 4.74 years with ages ranging between 35 and 45 years; mean follow-up time is 10.6 ± 2.5 months. Myopia ranged between −9.5 and −24 diopters (mean −16.3 ± 5.07 diopters). Mean preoperative UCVA was 0.05 ± 0.01 and mean postoperative UCVA was 0.54 ± 0.14. Mean preoperative BCVA was 0.37 ± 0.12 while mean postoperative BCVA was 0.64 ± 0.24. Mean postoperative refraction was –0.76 ± 1.22 D. There was a significant difference between the pre- and postoperative BCVAs (*P* > 0.0001). Mean preoperative IOP was 14.23 ± 2.5 mmHg and postoperative IOP was 13.72 ± 3.1 mmHg which did not differ significantly (*P* = 0.1). The mean follow-up period was 11.76 ± 1.4 months (ranging from 10 to 12 months). We did not report any intraoperative complications, to either RLE or primary posterior capsulotomy. The IOLs remained well centered after creation of the capsulotomy. Postoperatively, there were no cases of pupillary capture or distortion and no lens decentration or dislocation during the follow-up period. No eye required YAG laser posterior capsulotomy (Figure 1). There were no cases of retinal detachment throughout the follow-up period.

## 3. Discussion

The principal finding in this study is that primary posterior capsulotomy created by vitrectomy probe during RLE is a simple technique to prevent the occurrence of PCO and the need for a second procedure of YAG laser posterior capsulotomy with its well-known complications.

Refractive lens exchange can address all types of refractive errors providing a more predictable results and rapid recovery [10]. The rationale for combining refractive lens exchange with primary posterior vitrectorhexis in highly myopic patients is based on a number of considerations. PCO is one of the common complications of lens surgery. Although PCO can be managed with YAG laser capsulotomy, this means another procedure with potential risks including retinal detachment. Lastly, surgical posterior capsulotomy is a relatively simple and easy technique and has been used routinely in pediatric cataract surgery. PCO also is one of the most common visually disabling complications of lens surgery which still develops and necessitates YAG capsulotomy despite all methods that can be used to reduce its incidence such as cortical cleaving hydrodissection, meticulous cortical clean up, and implantation of a sharp posterior edge IOL [4, 11].

However, PCO was still reported even with improvement in IOL biomaterials with a reported incidence of 8.7% and 10.7% using acrylic lenses [12, 13].

During a 7-year follow-up period, Colin et al. 1999 reported 61.2% incidence of PCO necessitating capsulotomy after CLE for high myopia [14]. Fernandez-Vega et al. reported that the Nd-YAG laser capsulotomy risk was 77.89% following RLE for the correction of high myopia [15]. Another study reported only 4.2% incidence of PCO after clear lens extraction for severe myopia after a 15-month follow-up period [16]. YAG capsulotomy is relatively an easy procedure but it should never give to patient or physician the impression that it is risk free. Many studies have reported increased RD rates (0.5% to 3.6%) after Nd-YAG capsulotomy [17, 18]. Other studies did not indicate a major increased risk of RD after YAG capsulotomy [19, 20]. Each millimeter of increased axial length increases the risk of RD after YAG capsulotomy by a factor of 1.5 [7]. The exact mechanism of RD after laser capsulotomy is not well established. Laser energy may induce vitreous liquefaction, PVD or both, which might induce new breaks or enable preexisting breaks to progress to RD [7, 21].

Considering the young age of RLE patients, a relatively recent study reported that laser energy levels required for posterior capsulotomy were found to be slightly higher in younger subjects. In addition, there was a significant correlation between complications of YAG capsulotomy and laser energy levels [5]. This is an additional reason for caution in considering a simple technique as YAG laser posterior capsulotomy in cases with posterior capsular opacification in young highly myopic patients. Therefore, in our study we aimed to avoid the use of YAG laser capsulotomy by preventing PCO through doing primary posterior vitrectorhexis. Surgical capsulotomy is not a new technique. It was used to treat PCO before introducing Nd-YAG laser capsulotomy by Aron-Rosa in 1980 [22]. Surgical capsulectomy and anterior vitrectomy was also advised if extensive lens material or dense fibrosis prevents the safe use of YAG laser capsulotomy [23]. Janknecht & Funk reported that surgical peeling and aspiration of pearls might be a better alternative to YAG capsulotomy in myopic eyes [24]. Primary posterior capsulotomy combined with anterior vitrectomy is a well-known procedure in pediatric cataract surgery irrespective of their refractive status. Wilson et al., and Hazirolan et al., concluded that vitrectorhexis is well suited for use in children for anterior and posterior capsulorhexis [25, 26].

Using this technique dose not add an extra cost as most of modern phacomachines have a built-in anterior vitrectomy module. In addition, we decreased the financial burden of doing postoperative YAG laser capsulotomy. Our study has some limitations: the number of the patients is not sufficiently high, the control group is lacking, and there is a relatively short follow-up period. Future studies with more patients and a longer follow-up period might be required to get a more definite conclusion.

## 4. Conclusion

Vitrectorhexis is easy to learn, more predictable, and reproducible. Moreover, as we performed vitrectorhexis after IOL implantation, there will be less risk of posterior rhexis extension during IOL implantation. Primary posterior vitrectorhexis during RLE is an efficient method in preventing the occurrence of posterior capsular opacification (PCO) and the need for YAG laser posterior capsulotomy with its possible complications.

## Supplementary Material

Video 1: Surgical steps of clear lens extraction combined with posterior vitrectorhexis.
